# Effect of neuroanatomy on corticomotor excitability during and after transcranial magnetic stimulation and intermittent theta burst stimulation

**DOI:** 10.1002/hbm.25968

**Published:** 2022-06-09

**Authors:** Neil Mittal, Bhushan Thakkar, Cooper B. Hodges, Connor Lewis, Yeajin Cho, Ravi L. Hadimani, Carrie L. Peterson

**Affiliations:** ^1^ Department of Biomedical Engineering Virginia Commonwealth University Richmond Virginia USA; ^2^ College of Engineering Virginia Commonwealth University Richmond Virginia USA; ^3^ Department of Physical Therapy Virginia Commonwealth University Richmond Virginia USA; ^4^ Department of Physical Medicine and Rehabilitation Virginia Commonwealth University Richmond Virginia USA; ^5^ Department of Mechanical and Nuclear Engineering Virginia Commonwealth University Richmond Virginia USA

**Keywords:** finite element analysis, magnetic resonance imaging, motor evoked potentials, neuroanatomy, neuroplasticity, pyramidal tract (corticospinal), rehabilitation

## Abstract

Individual neuroanatomy can influence motor responses to transcranial magnetic stimulation (TMS) and corticomotor excitability after intermittent theta burst stimulation (iTBS). The purpose of this study was to examine the relationship between individual neuroanatomy and both TMS response measured using resting motor threshold (RMT) and iTBS measured using motor evoked potentials (MEPs) targeting the biceps brachii and first dorsal interosseus (FDI). Ten nonimpaired individuals completed sham‐controlled iTBS sessions and underwent MRI, from which anatomically accurate head models were generated. Neuroanatomical parameters established through fiber tractography were fiber tract surface area (FTSA), tract fiber count (TFC), and brain scalp distance (BSD) at the point of stimulation. Cortical magnetic field induced electric field strength (EFS) was obtained using finite element simulations. A linear mixed effects model was used to assess effects of these parameters on RMT and iTBS (post‐iTBS MEPs). FDI RMT was dependent on interactions between EFS and both FTSA and TFC. Biceps RMT was dependent on interactions between EFS and and both FTSA and BSD. There was no groupwide effect of iTBS on the FDI but individual changes in corticomotor excitability scaled with RMT, EFS, BSD, and FTSA. iTBS targeting the biceps was facilitatory, and dependent on FTSA and TFC. MRI‐based measures of neuroanatomy highlight how individual anatomy affects motor system responses to different TMS paradigms and may be useful for selecting appropriate motor targets when designing TMS based therapies.

## Graphical abstract

Individual neuroanatomy can influence motor responses to transcranial magnetic stimulation (TMS) and corticomotor excitability after intermittent theta burst stimulation (iTBS). Parameters of individual neuroanatomy as measured from individually accurate MRI‐derived head models were compared to empirical measures of TMS response and iTBS effects. These MRI‐based measures of neuroanatomy showed that individual anatomy affects motor system responses to TMS and may be useful for selecting motor targets when designing TMS based therapies.

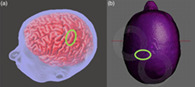

## 1 INTRODUCTION

Transcranial magnetic stimulation (TMS) techniques have received increased attention in recent years as potential treatments for neurological disorders via manipulating cortical excitability, such as traumatic brain injury, stroke, spinal cord injury, and movement disorders (Bender Pape et al., 2020; Korzhova et al., 2019; Pink et al., 2021; Spagnolo et al., 2021). Intermittent theta burst stimulation (iTBS) is a form of repetitive transcranial magnetic stimulation (rTMS) that can facilitate corticomotor excitability (Butts et al., 2014; Hinder et al., 2014; Huang et al., 2005; Klomjai et al., 2015; Suppa et al., 2016). Motor targets investigated in iTBS studies include the biceps brachii (Mittal et al., 2021) and the first dorsal interosseous (FDI) (Butts et al., 2014; Hinder et al., 2014; Huang et al., 2005; Klomjai et al., 2015; Suppa et al., 2016) due to their roles in rehabilitation and activities of daily living (ADLs). iTBS has been evaluated in individuals with spinal cord injury (SCI), showing variable effects (Fassett et al., 2017). Other work has noted that although TMS techniques are therapeutically promising, considerable work remains to be done in determining the driving factors behind treatment response variability (Bender Pape et al., 2020).

High variability has been reported in changes in corticomotor excitability both within and across individuals in TMS studies (Guerra et al., 2020; Hamada et al., 2013; Hinder et al., 2014; Huang et al., 2017; López‐Alonso et al., 2014; Nettekoven et al., 2015; Perellón‐Alfonso et al., 2018). This is seen regarding response to TMS as measured by motor thresholds (MT) (Perellón‐Alfonso et al., 2018; Sollmann et al., 2016), and response to iTBS as measured by motor evoked potentials (MEPs) (Cárdenas‐Morales et al., 2014; Corp et al., 2020; Darling et al., 2006; López‐Alonso et al., 2014; Perellón‐Alfonso et al., 2018). Factors contributing to TMS motor threshold variability include individual differences of synaptic plasticity (Perellón‐Alfonso et al., 2018; Ridding & Ziemann, 2010), use of medication, clinical pathology, age, and gender (Sollmann et al., 2016). Factors contributing to the variability (Suppa et al., 2016) of iTBS effects include genetics (Jannati et al., 2017), age (Ferreri et al., 2017), motor target (Malcolm et al., 2006), cortical organization (Cárdenas‐Morales et al., 2014; Hamada et al., 2013), alertness (Noreika et al., 2020), neurotransmitter and receptor variation (Ziemann et al., 1996, 2015), and brain anatomy (Syeda, Magsood, et al., 2017). Individual neuroanatomy can also contribute to variability (Lee et al., 2016). Distinct motor regions in the brain have unique characteristics, such as surface area (Malcolm et al., 2006), neuron density (Darling et al., 2006), or orientation of neurons with respect to the skull (De Geeter et al., 2015). However, the effects of these individual level differences are not well characterized.

Intrinsic variability of TMS metrics is an important confounder in TMS studies. MEPs are associated with high variability that frequently remains uncontrolled for within study designs, stemming from sources previously mentioned, as well as physiological influences including, but not limited to, constant fluctuations in the corticospinal excitability of neurons at both the corticospinal and segmental level (Kiers et al., 1993) and the influence of the whole corticospinal tract (Rösler et al., 2008).

Individual neuroanatomy would be expected to affect responsiveness to iTBS because the conduction of the induced current from TMS is dependent on the morphology and material properties of the stimulated medium, and individual brains have unique anatomical features. Furthermore, anatomical complexities of cortical motor regions and their corresponding fiber tracts determine TMS induced electric fields in the brain, which are the mechanistic impetus for TMS activation and more specifically rTMS paradigms (Di Lazzaro et al., 2008; Huang et al., 2005). Depolarization of the neurons in the motor cortex elicits responses to TMS, and as a result, brain anatomy and related morphology features likely impact the effectiveness of iTBS even for different motor targets of the same individual (Cárdenas‐Morales et al., 2014; Hamada et al., 2013; Lee et al., 2016, 2018; Malcolm et al., 2006; Syeda, Magsood, et al., 2017). For example, brain scalp distance (BSD) has been associated with TMS response (Crowther et al., 2014; Lee et al., 2016). However, BSD is limited as it is a one‐dimensional parameter as opposed to induced electric field which is a three dimensional parameter that takes into account the composition of tissue between the scalp and cortex (Syeda, Magsood, et al., 2017).

The objectives of this preliminary study were to determine the effects of individual brain neuroanatomy as measured by simulated induced electric fields from TMS over motor cortical regions of the biceps and FDI on: a) resting motor threshold (RMT) and b) MEPs after iTBS. Healthy individuals underwent iTBS for empirical data collection and magnetic resonance imaging (MRI), from which we developed anatomically accurate computational head models with relevant neuroanatomy (Lee et al., 2016; Syeda, Magsood, et al., 2017). Induced electric fields were computed in head models using finite element analysis across individual brain morphology; fiber tract geometry was determined based on surface area and fiber count. The neurophysiological effects of single pulse TMS and iTBS in the form of RMT and MEPs, respectively, were recorded in the same participants. Our central hypothesis was that brain anatomy evidenced by the simulated induced electric field would influence corticomotor excitability. First, we hypothesized that empirically derived RMT would negatively correlate with the magnitude of the simulated induced electric field and with fiber tract size, regardless of the motor target. The basis of this hypothesis was that a greater responsiveness to TMS (as indicated by lower RMT) would relate to a larger induced cortical current (and corresponding white matter tract). This would establish a relationship between model‐derived parameters and empirical single pulse TMS response. Second, we hypothesized that individuals with greater simulated electric field strength would exhibit a larger change in corticomotor excitability (as measured by increased MEP amplitude) after iTBS in both motor targets. Lastly, we hypothesized that the specific factors such as electric field strength and fiber tract geometry would differently influence the response from the two motor targets (biceps and FDI) due to differences in cortical architecture. Overall, we sought to elucidate whether MRI‐based measures of neuroanatomy can predict whether an individual is likely to respond to iTBS‐based therapies.

## 2 METHODS

### 2.1 Participants

Ten healthy individuals (7 females and 3 males, 23.5 ± 5 years) participated in this study (Tables 1 and 2). The inclusion criteria required participants to be between the ages of 18 and 65 years old. Exclusion criteria were presence of severe medical illness and sequelae, existing infection, cardiovascular disease, significant osteoporosis, metal implanted devices, personal or family history of seizure activity, and any acute or current history of neuromuscular or motor dysfunction. All participants were screened to ensure safety of the TMS and MRI protocols and provided informed consent. This study was approved by the Virginia Commonwealth University Institutional Review Board.

**TABLE 1:** Motor thresholds and maximum voluntary contraction (MVC) prior to first dorsal interosseus (FDI) iTBS presented as mean *±* one standard deviation

			Prior to sham iTBS			Prior to active iTBS		
Participant	Age	FDI MVC EMG^a^ (mV)	FDI RMT^b^	FDI AMT^c^	Average baseline nMEPs^d^	FDI RMT	FDI AMT	Average baseline nMEPs
01	21	238.3	46	38	0.353	49	35	0.660
02	24	472.4	41	48	0.211	41	47	0.295
03	19	170.1	68	31	0.735	55	36	0.351
04	20	130.7	64	43	0.713	64	50	0.727
05	23	504.7	72	28	0.034	78	40	0.369
06	19	750.4	70	36	0.250	50	50	0.045
07	32	263.3	43	37	0.134	43	44	0.113
08	26	370.6	41	33	0.404	33	42	0.320
09	29	887.9	54	30	0.136	51	39	0.399
10	27	740.9	70	39	0.170	68	47	0.502
Mean ± SD	23.5 ± 5	452.93 ± 265.9	56.9 ± 13	36.3 ± 6	0.2941 ± 0.269	53.2 ± 14	43.0 ± 5	0.3891 ± 0.314

^a^ MVC: maximum voluntary contraction.

^b^ RMT: resting motor threshold as percent of maximum stimulator output (% MSO) measured with monophasic stimulation inducing an AP current in the brain.

^c^ AMT: active motor threshold as % MSO measured with biphasic PA/AP stimulation.

^d^ nMEP: normalized motor evoked potential (%MVC) measured with monophasic AP stimulation, presented as mean ± one standard deviation.

**TABLE 2:** Motor thresholds and maximum voluntary contraction (MVC) prior to biceps iTBS presented as mean *±* one standard deviation

			Prior to sham iTBS			Prior to active iTBS		
Participant	Age	Biceps MVC EMG^a^ (mV)	Biceps RMT^b^	Biceps AMT^c^	Average baseline nMEPs^d^	Biceps RMT	Biceps AMT	Average baseline nMEPs
01	21	411.0	57	68	0.066	61	68	0.059
02	24	118.0	62	60	0.067	67	58	0.112
03	19	183.2	100	70	0.011	100	74	0.011
04	20	144.6	49	67	0.024	65	74	0.032
05	23	185.2	86	72	0.041	87	73	0.025
06	19	453.2	61	70	0.136	85	48	0.102
07	32	184.1	100	72	0.023	100	73	0.012
08	26	128.9	69	57	0.078	78	52	0.12
09	29	256.0	44	44	0.093	47	46	0.292
10	27	112.2	100	62	0.065	100	65	0.055
Mean ± SD	23.5 ± 5	217.63 ± 121.1	72.8 ± 22	64.2 ± 9	0.0602 ± 0.049	79.0 ± 19	63.1 ± 11	0.0820 ± 0.084

^a^ MVC: maximum voluntary contraction.

^b^ RMT: resting motor threshold as percent of maximum stimulator output (% MSO) measured with monophasic stimulation inducing an AP current in the brain.

^c^ AMT: active motor threshold as % MSO measured with biphasic PA/AP stimulation.

^d^ nMEP: normalized motor evoked potential (%MVC) measured with monophasic AP stimulation, presented as mean ± one standard deviation.

### 2.2 Experiment overview

**FIGURE 1:**
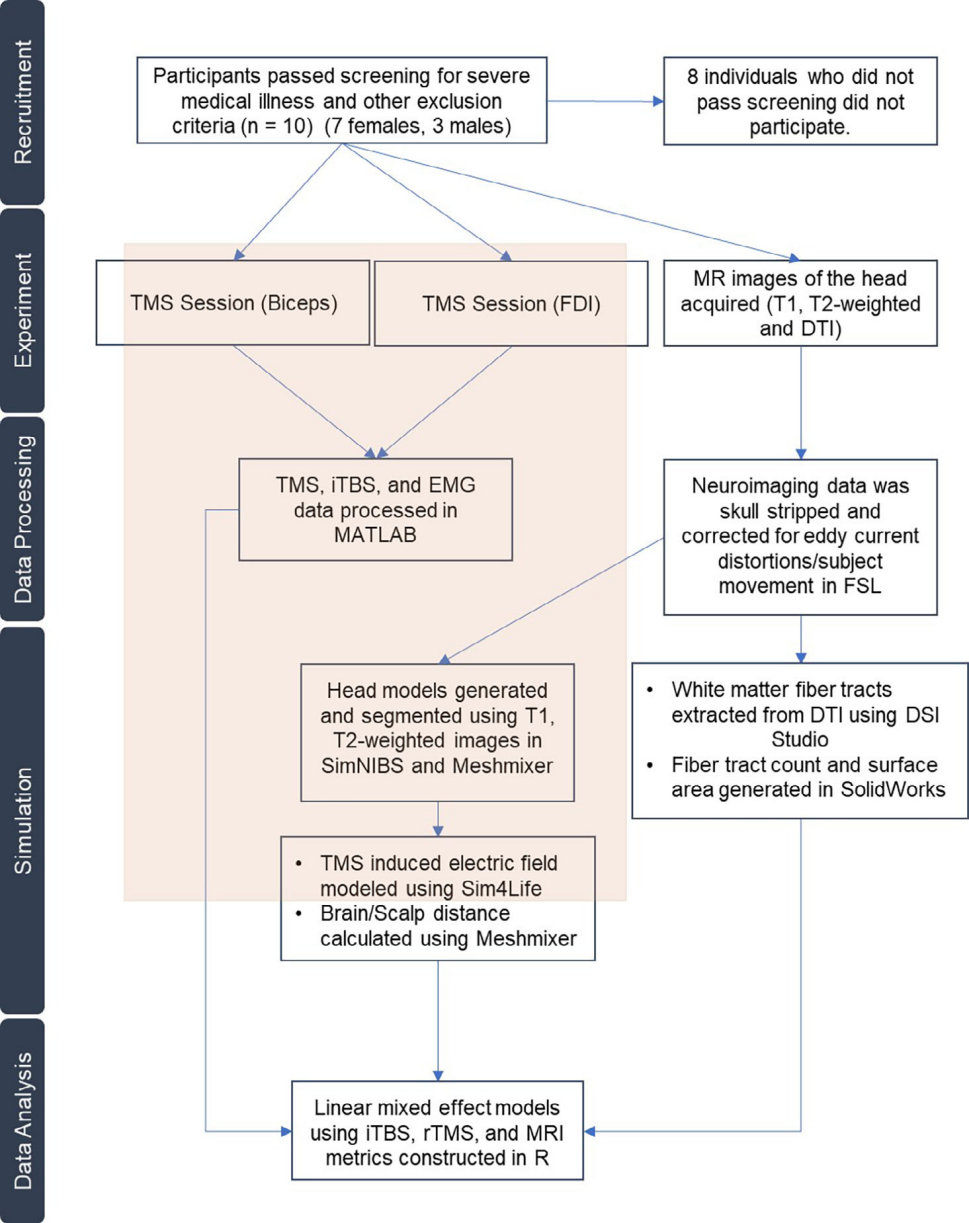
Experimental design. Participants underwent two sessions of TMS for empirical measurements (FDI and biceps), and one MRI to develop individualized, neuroanatomically accurate simulations. MRI head images were used to establish neuroanatomical parameters for each participant; these parameters were evaluated for their influence on empirical TMS data. The shaded region represents empirical and simulated TMS data.

Each participant completed one FDI targeted iTBS session, one biceps targeted iTBS session, and an MRI session of the head on three separate days. The iTBS sessions were separated by a minimum of 3 days to prevent carry over effects (Huang et al., 2017). Sessions were scheduled for the same time of day for each participant to control for diurnal effects. MRI data were used to generate head models for neuroanatomical parameters. The experimental steps can be seen in Figure 1.

### 2.3 Transcranial magnetic stimulation experiments

**FIGURE 2:**
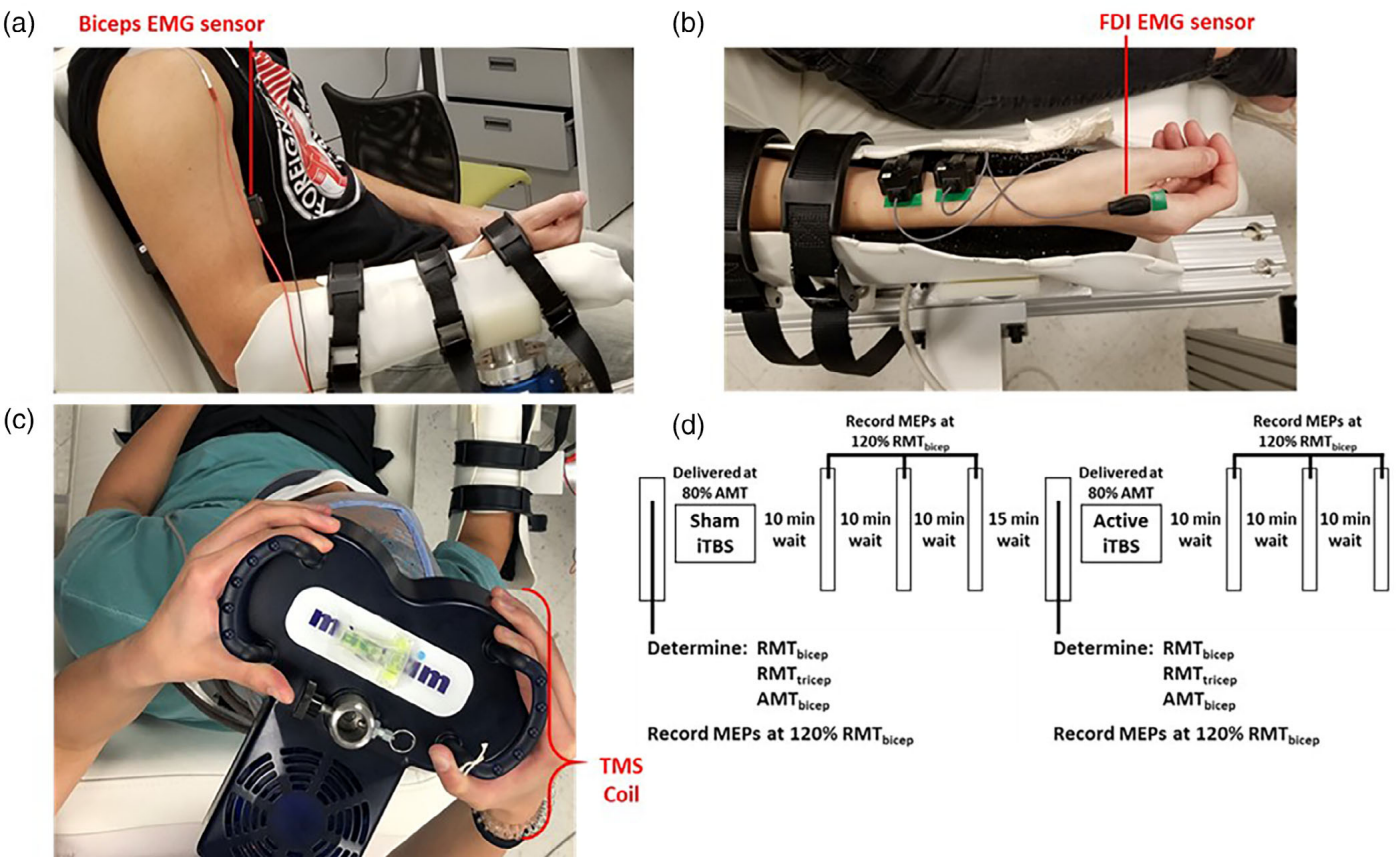
Experimental setup and structure of TMS sessions. (a, b) Participants' forearms were supported in the horizontal plane with EMG sensors placed on their biceps (a) or first dorsal interosseus (FDI) (b). (c) The TMS coil was placed tangentially over the scalp above the motor cortex, oriented to induce a posterior–anterior current within in the motor cortex. (d) Sessions began with motor threshold and baseline MEP measurements before performing iTBS. MEPs were collected post‐iTBS at 120% RMT in 10‐min intervals. Sham iTBS did not deliver active stimulation.

Electromyography (EMG) data were recorded using a Trigno™ Wireless System (Delsys, Natick, MA). EMG signals were recorded with Spike 2 software (Cambridge Electron Design, Cambridge, UK). The FDI and first palmer interosseus (FPI) for FDI sessions, and long head of the biceps and the lateral head of the triceps for biceps sessions were instrumented with surface electrodes on the skin, verified by functional muscle testing (Figure 2). FPI and triceps were instrumented for monitoring purposes. EMG signals were amplified (×1000), bandpass‐filtered (20–450 Hz) prior to A/D conversion (Micro 1401 MkII, Cambridge Electron Design, Cambridge, UK), and sampled at 2000 Hz.

Single pulse TMS was delivered as a monophasic posterior–anterior current to the primary motor cortex contralateral to the resting arm using a Magstim BiStim^2^ stimulator via a 70‐mm figure‐of‐eight coil (P/N 4150‐00). iTBS was performed using a Magstim Super Rapid^2^ Plus^1^ stimulator (Magstim, Whitland, UK) via a 70‐mm figure‐of‐eight air film coil (P/N 3910–00) that delivered high‐frequency biphasic pulses with currents in the posterior–anterior then anterior–posterior directions. The vertex at the intersection of the inion‐nasion and inter‐aural lines were marked on a cap fitted on the participant's head and used to orient the coil near the cortical target. The coil was held tangentially on the scalp via a support stand with the coil center rotated to induce a posterior‐to‐anterior cortical current across the central sulcus (Figure 2c). The hotspot for the target muscle was identified as the location evoking the largest peak‐to‐peak amplitude MEP using the lowest stimulation intensity (Ah Sen et al., 2017; Jung et al., 2010).

RMT was defined as the lowest stimulus intensity that induced MEPs of ≥50 μV in at least 5 of 10 consecutive stimuli with the target muscle fully relaxed (Borckardt et al., 2006). AMT was defined as the stimulus intensity that elicited a MEP of ≥200 μV in at least 5 of the 10 consecutive stimuli recorded during sustained isometric contraction of 10 ± 5% of the participant's maximum effort (Borckardt et al., 2006; Hinder et al., 2014). Maximum effort was measured by the average EMG in the highest 0.5 s period of a 5‐s isometric maximum voluntary contraction (MVC), averaged across 3 trials. Stimulus intensity was determined using an adaptive parameter estimation by sequential testing software (Borckardt et al., 2006). Evoked potential operant conditioning software (EPOCS) developed by the Evoked Potential Operant Conditioning Core at the National Center of Neuromodulation for Rehabilitation was used to record motor thresholds and display effort levels for participants.

iTBS was applied using a Magstim Super Rapid^2^ Plus^1^ stimulator and a 70‐mm double air film coil following the common protocol presented by Huang et al. applied to motor areas (Y. Huang et al., 2005) (Figure 2d). iTBS applied to the motor target cortical hotspot consisted of three pulses presented at 50 Hz, every 200 ms for 2 s, for 8 s, at a subthreshold intensity of 80% of the participant's AMT resulting in 600 pulses (Y. Huang et al., 2005; Suppa et al., 2016). During sham iTBS, a Magstim 70‐mm figure‐of‐eight air film sham coil (P/N: 3950–00) (Magstim, Whitland, UK) was used which looked identical to the active coil and made similar noises without delivering any stimulation (Harvey et al., 2018; McGirr et al., 2021; Mittal et al., 2021). Throughout each session participants were blinded to the type of stimulation they were receiving.

**FIGURE 3:**
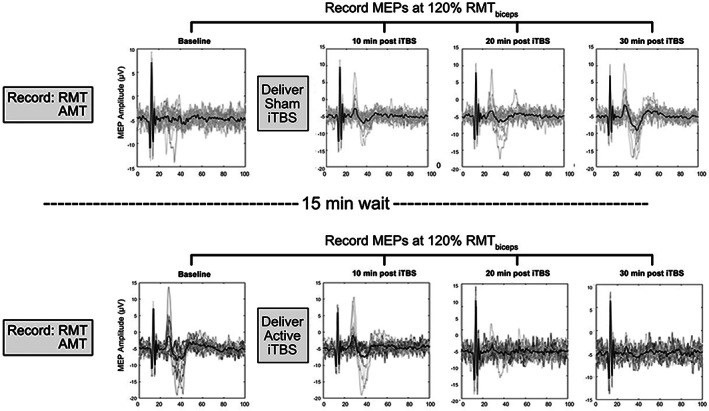
TMS sessions and empirical data. Before application of iTBS, single pulse TMS was used to determine RMT, AMT, and collect baseline MEPs for the motor target. iTBS was delivered at an intensity of 80% of AMT. Single pulse TMS elicited MEPs at 10‐min intervals following iTBS, at 120% RMT. Data shown represent processed and collected raw EMG from which MEPs were calculated of a single session from a representative participant. Gray lines represent individual MEPs and the black line represents the average MEP. Horizontal axis depicts time (ms).

Participants received single pulse TMS to elicit MEPs before iTBS and in 10‐min intervals after for 30 min (Figure 3). During each time interval, no more than 20 stimulations were delivered.

### 2.4 TMS data processing

Using purpose‐written code in MATLAB (MathWorks, MATLAB v 9.7.0.1190202), peak‐to‐peak MEP amplitudes were calculated from the motor target EMG data of each session. The root mean square (RMS) amplitude was calculated for the evoked response over a 50‐ms window (12–62 ms post TMS pulse), and for a 50‐ms window prior to the TMS pulse (pre‐stimulus). Instances where the pre‐stimulus RMS exceeded the evoked response RMS were excluded (Darling et al., 2006). MEPs were then normalized by and presented as a percentage of the MVC EMG (Halaki & Gi, 2012). Normalized MEPs (nMEPs) served as our measure of corticomotor excitability, with the average of nMEPs collected prior to iTBS serving as the baseline.

### 2.5 Neuroimaging acquisition

Structural T1‐ and T2‐weighted images were acquired using a Philips 3.0T Ingenia system with a 32‐channel receive head coil (Philips Medical Systems, Best, Netherlands). T1‐weighted images were acquired using a 3D MPRAGE sequence with the following parameters: repetition time (TR) = 8 ms, echo time (TE) = 3.7 ms, acquired sagittally with a 1.0 × 1.0 × 1.0 mm resolution at a flip angle of 8°, echo train length (ETL) = 256, matrix = 256 × 240). T2‐weighted images were acquired using a 3D multishot turbo spin echo sequence (TE/TR = 245/2500 ms acquired sagittally with a matching resolution of the T1‐weighted images, two averages, flip angle = 90°, ETL = 117, matrix = 256 × 256).

Whole brain diffusion weighted images were acquired in the transverse plane using a single shot diffusion sensitized spin echo planar imaging sequence (Hasan & Narayana, 2003) with the following parameters: b‐factors = 1000 s/mm 2 and 0 s/mm 2, SENSE in‐plane acceleration factor = 2.75, repetition time (TR) = 6.05 seconds, echo time (TE) = 96 ms, half‐scan factor = 0.602, 60 diffusion directions, 6 repetitions of b‐factor = 0, field of view = 256 × 256, acquisition matrix = 140 × 141, slice thickness = 1.7 mm, 80 slices, flip angle = 90°, and a voxel resolution of 1.7 mm × 1.7 mm × 1.7 mm. The diffusion tensor imaging (DTI) acquisition time was approximately 10 min per subject.

### 2.6 Neuroimaging preprocessing

DTI images were transformed to Neuroimaging Informatics Technology Initiative format using dcm2niix (Li et al., 2016). DTI images were then preprocessed using the FMIRB Software Library (FSL, version 6.0) (Jenkinson et al., 2012). Images were corrected for eddy current‐induced and head motion‐induced distortions using a variant of the eddy command (Andersson & Sotiropoulos, 2016) called eddy_cuda8.0, which uses Compute Unified Device Architecture, an accelerated computing platform on NVIDIA graphics processor units, to parallelize analyses. Brain tissue was extracted and an exclusion mask generated using FSL's Brain Extraction Tool through the bet2 command (Smith, 2002). These corrected and extracted images were then used in subsequent head model generation. The workflow for MRI images and subsequent model derivations can be seen in Figure 3.

**FIGURE 4:**
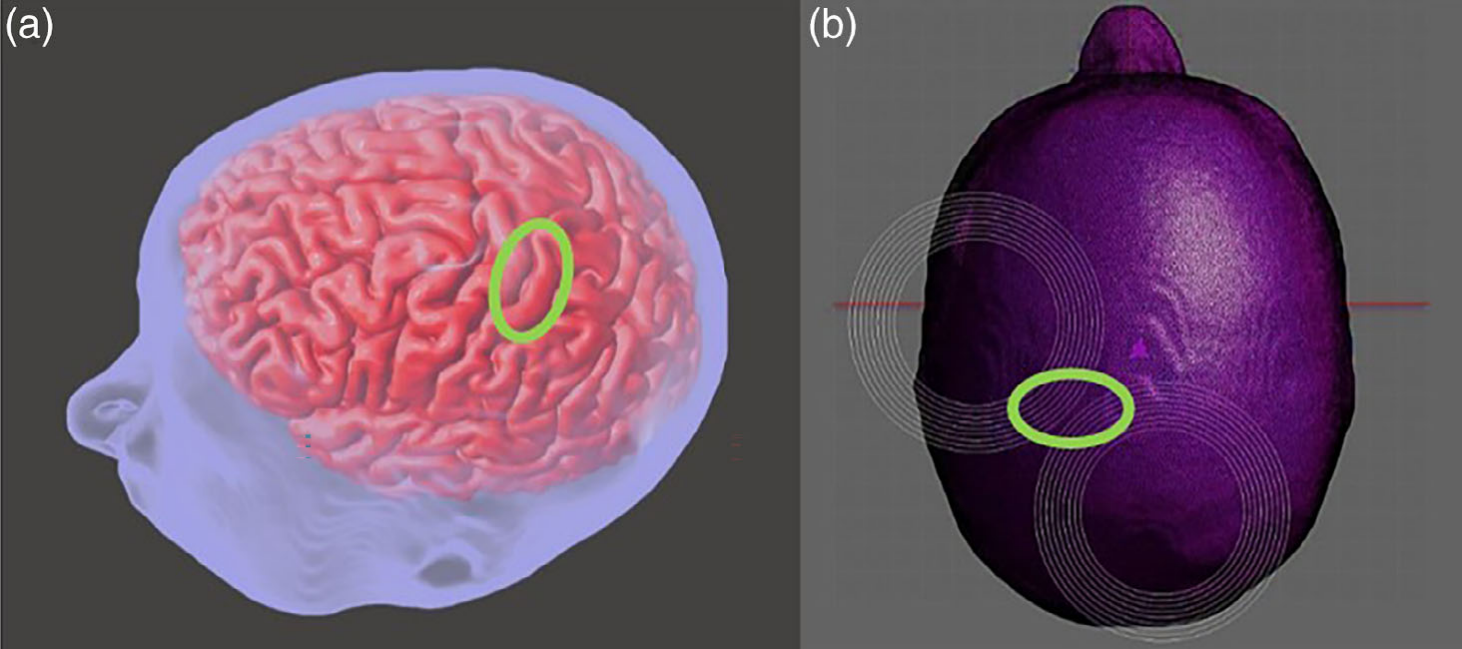
MRI‐derived individual anatomically accurate head models. (a) Head models integrated seven segments derived from MRI: skin (blue), bone (transparent), cerebrospinal fluid (transparent), gray matter (pink), ventricular space (below the visible gray matter), cerebellum (below the visible gray matter), and white matter (below the visible gray matter). The gray matter can be seen as the surface of the cortex in this image. (b) Head models then underwent finite element simulation of TMS. White concentric circles represent the modeled coil. The green circle (a,b) encompasses the hand knob representative of upper limb control of the motor cortex.

Using the extracted T1‐ and T2‐weighted images from all the subjects, a SimNIBS pipeline (SimNIBS Developers 2019, v2.0.1) (Lee et al., 2016, 2018) was used to create seven separate segments (white matter, gray matter, cerebrospinal fluid, skin, skull, ventricles, and cerebellum) as separate 3D modeled files. Abnormalities were smoothed using Meshmixer (AutoDesk, Inc. v11.2.37) (Figure 4a).

### 2.7 Induced electric field modeling

Induced electric field from peak intensity stimulation at the upper limb control region of the primary motor cortex, was computed using Sim4Life finite element analysis software(Zurich Med Tech, v6.2.1.4972) (Syeda, Pandurangi, et al., 2017), on the generated head models (Crowther et al., 2014; Lee et al., 2016). Head model segments were imported into Sim4Life (Syeda, Pandurangi, et al., 2017). The simulated coil was defined to match dimensions and function of the Magstim 70‐mm figure‐of‐8 coil (De Deng et al., 2012). The coil was oriented with the center directly over the region of stimulation interest with 45° orientation to the coronal plane to match the empirical test setup (Figure 4b). The target region of stimulation was identified as the precentral gyrus posterior to the superior frontal sulcus, within the “knob” as defined by Yousry and consistent with approximations from motor homunculi (Yousry et al., 1997).

**FIGURE 5:**
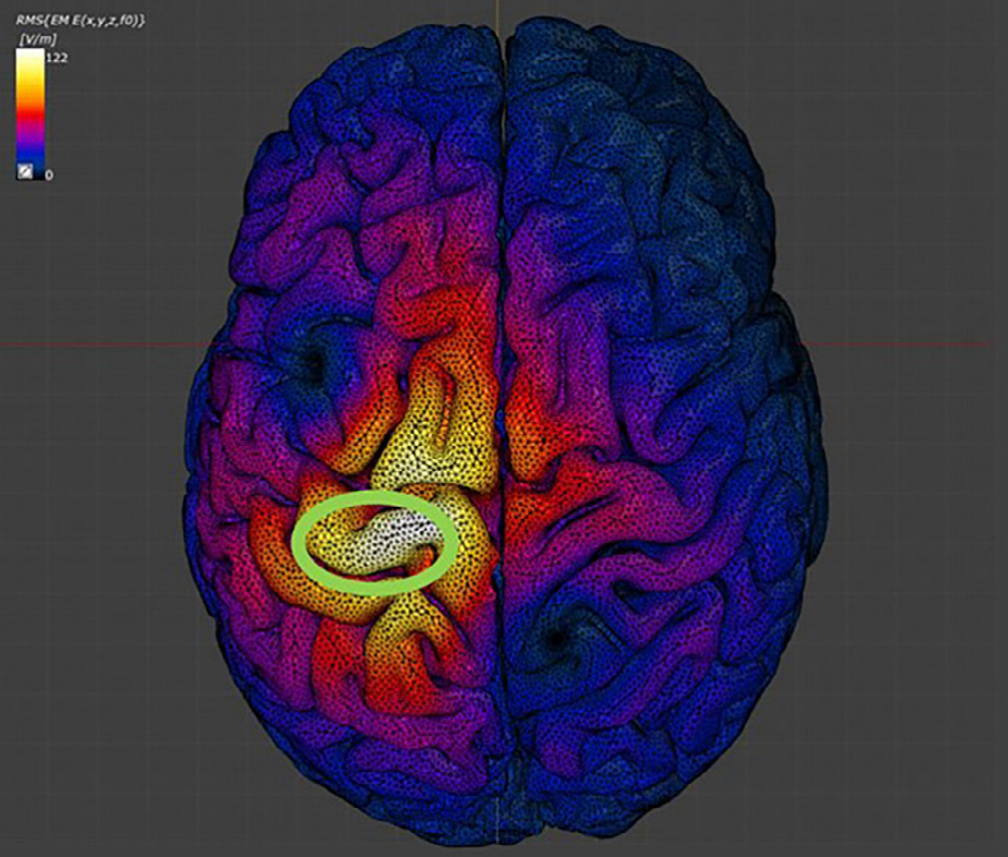
Induced electric field strength. Finite element simulations of the individual head models were performed to calculate the induced electric field strength of magnetic stimulation of the motor cortex. Maximal stimulation was confirmed to be over the primary motor cortex in the region of upper limb control. Color bar represents induced electric field intensity (V/m), ranging from maximal (white) to minimal (dark blue). The green circle encompasses the hand knob representative of upper limb control of the motor cortex.

The stimulation current strength was set to 5000 A, corresponding to 100% MSO, at 2500 Hz (Syeda, Magsood, et al., 2017) and the seven segments of the head model as well as the air were assigned their respective material properties based on the IT'IS LF database (IT'IS Foundation, v4.0). The magnetic stimulation induced electric field strength (EFS) at the surface of the cortex at the point of interest, specifically of the gray matter segment, was determined (Figure 5). EFS was used in analyses to represent the simulated cortical current across the MRI‐derived anatomy for each individual.

Brain scalp distance (BSD) calculations were made using the gray matter and skin files from each subject in Meshmixer (Figure 4a). Calculations were made from identification of the surface of both segments using the same location that was designated as the target for the induced electric field simulations. BSD was used in analyses as a representation of magnetic field attenuation, due to the distance between the cortex and the coil surface.

**FIGURE 6:**
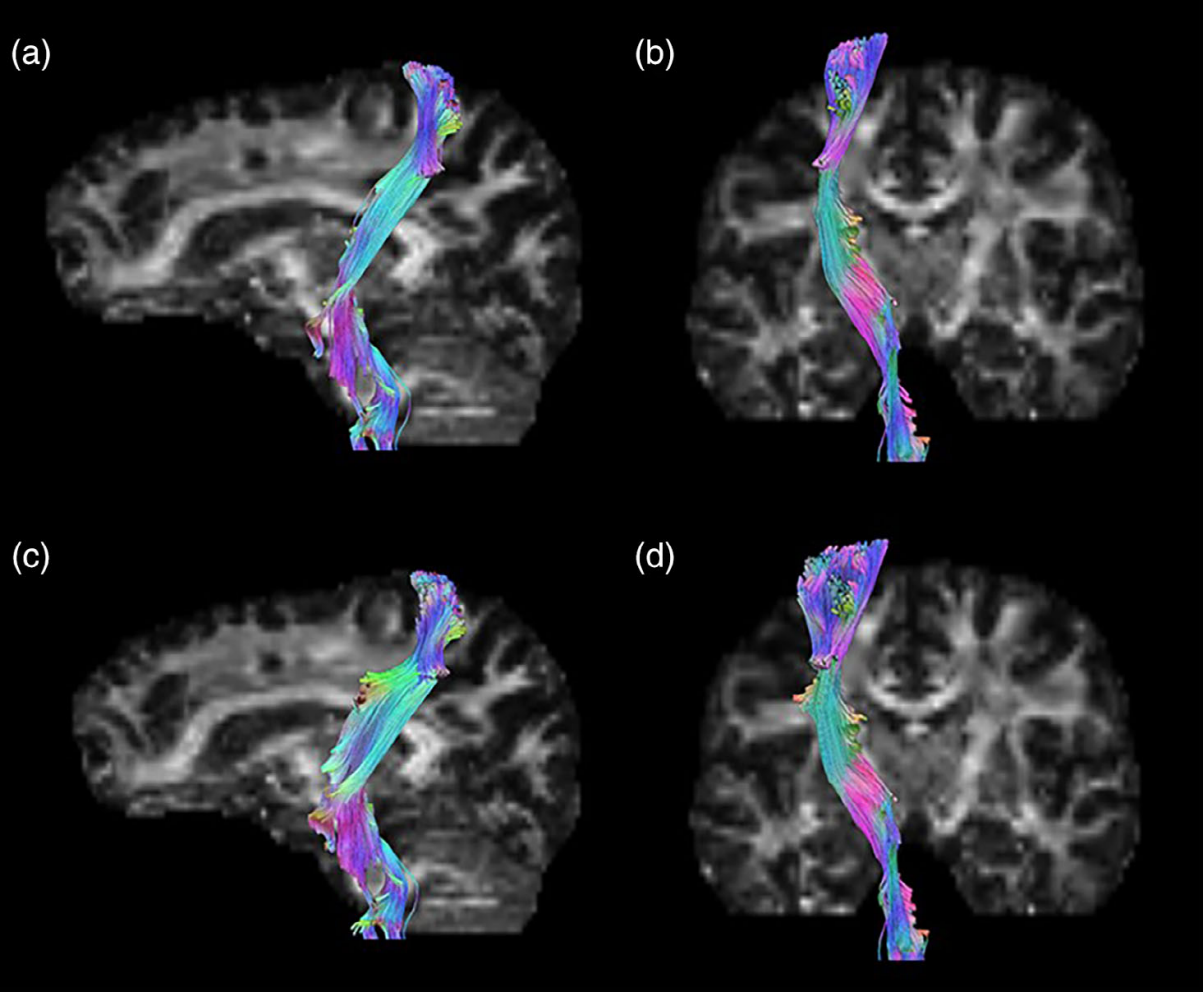
Fiber tractography. Regions of interest in the primary motor cortex representing the biceps (a, b) and FDI (c, d) were used to extract fiber tracts for upper limb motor control. Colorized tracts represent the pathways included after trimming for non‐corticospinal and recursive connections. (a) Biceps tracts, sagittal section. (b) Biceps tracts, coronal section. (c) FDI tracts, sagittal section. (d) FDI tracts, coronal section.

Fiber tracts were extracted from DTI data using DSI Studio (Feh, 2020). The left side of the brain was located, and the anatomical landmarks to the “knob” of the primary motor cortex (superior frontal sulcus, precentral sulcus, central sulcus, and precentral gyrus), were identified (Yousry et al., 1997). The region of interest was drawn using the circle tool with a 6 mm diameter to ensure gyrus coverage without extending beyond the precentral gyrus. The FDI region was drawn between the landmark sulci, centered on the precentral gyrus, and in line with the superior frontal sulcus along the “knob” region (Yousry et al., 1997). The biceps region was drawn medially to the FDI region, within the automated left corticospinal tract from DSI Studio. After the regions of interest were drawn, the fibers in the respective regions were extracted and trimmed following the automated corticospinal tract (Mak et al., 2018) (Figure 6). Fiber coordinates were then imported into SolidWorks (BIOVIA, Dassault Systèmes, SolidWorks, SP3.0, San Diego: Dassault Systèmes, 2017) and used to generate tract fiber counts (TFC) and fiber tract surface areas (FTSA) for each individual fiber tract. FTSA and TFC were used in analyses as representations of fiber tract size.

### 2.8 Statistical analysis

Linear mixed effects models were analyzed to test the effect of neuroanatomy on the empirical response to single pulse TMS, as measured by RMT. RMT served as the dependent variable, with model fixed effects of induced electric field, fiber tract surface area, tract fiber count, and BSD. Participants were included in the statistical model as a random effect because each TMS session collected two RMT values, one for MEP elicitation to study sham iTBS and one for active iTBS. Interactions between electric field and fiber tract geometry were considered. Linear mixed effects models were created and analyzed using purpose‐written R (The R Foundation, 2018) code based on the LME4 package (Bates et al., 2015; The R Foundation, 2018).

Linear mixed effects models were also analyzed to test the effect of neuroanatomy on nMEPs recorded after iTBS. The change from baseline and effect of stimulation type, sham or active, was used to assess effect of iTBS by the interaction between these parameters, and RMT was used as an input to represent empirical responsiveness to TMS. This assessment was performed separately for biceps and FDI motor targets.

## 3 RESULTS

For all participants, baseline measurements taken from TMS sessions are presented in Tables 1 and 2. Data supporting our results can be found through the Open Science Framework—https://osf.io/qtxn4/?view_only=54a0e81eeb3e44f8a90971cbbd07c433.

### 3.1 Effect of neuroanatomy on empirically measured RMT of the FDI

**FIGURE 7:**
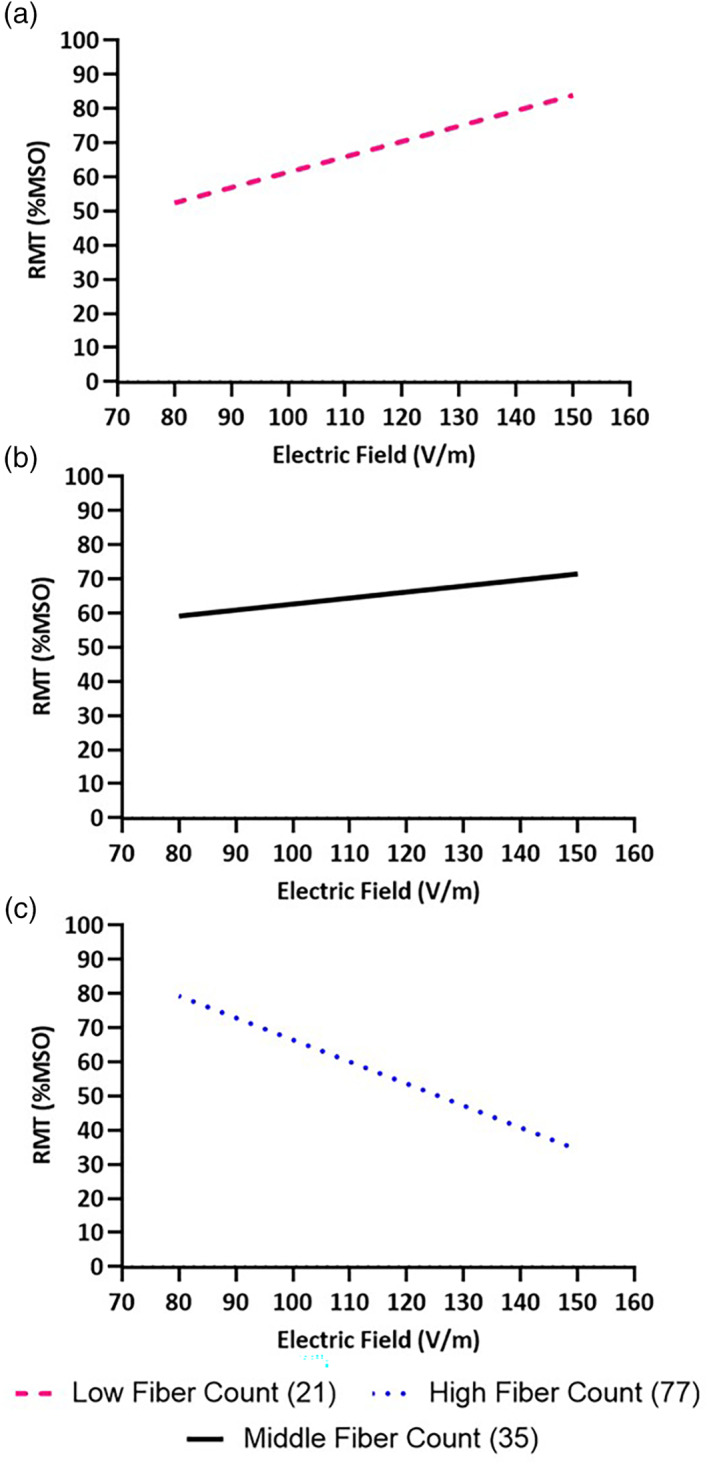
Effect of tract fiber count (TFC) on empirically measured RMT of the FDI. (a) At lower TFC, and (b) as TFC increased, RMT was positively correlated with electric field strength (EFS). (c) At higher TFC, RMT was less affected by EFS, evidenced by a simulated reversal of the relationship.

**FIGURE 8:**
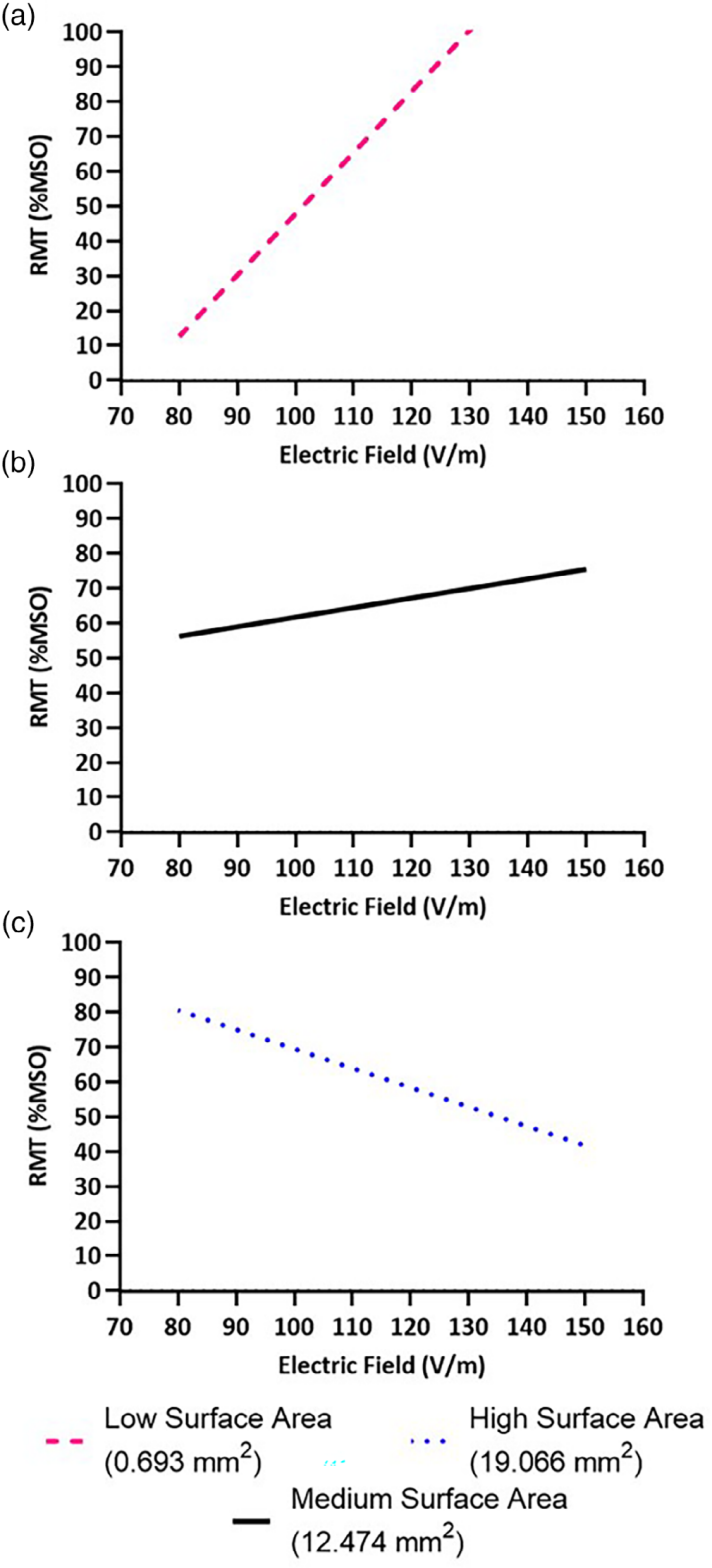
Effect of fiber tract surface area (FTSA) on empirically measured RMT of the FDI. (a) At lower FTSA, and (b) as FTSA increased, RMT was positively correlated with electric field strength (EFS). (c) At higher FTSA, RMT was less affected by EFS, evidenced by a simulated reversal of the relationship.

The FDI RMT correlated with the interaction between EFS and TFC in the FDI (χ2 = 8.14, p = .004). The FDI RMT also correlated with the interaction between EFS and FTSA (χ2 = 4.41, p = .036). BSD did not affect FDI RMT (*χ* ^2^ = 0.64, *p* = .423). Significant relationships are shown in Figures 7 and 8.

### 3.2 Effect of neuroanatomy on empirically measured RMT of the biceps

**FIGURE 9:**
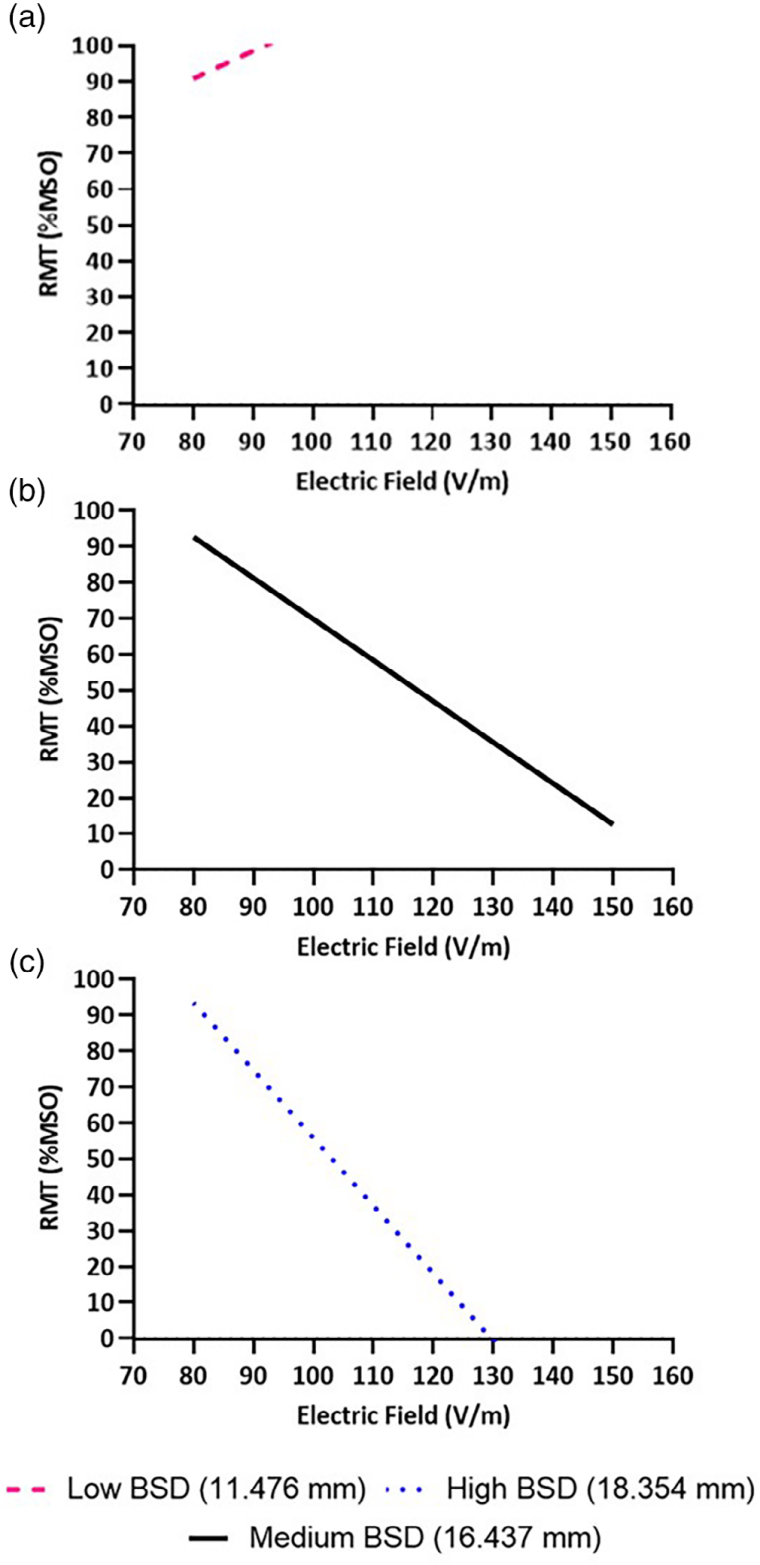
Effect of brain scalp distance (BSD) on empirically measured RMT of the biceps. (a) At lower BSD, RMT was not dependent on electric field strength (EFS), as evidenced by the reversal of the simulated relationship seen at other BSD magnitudes and calculated RMT's greater than 100% MSO. (b) As BSD increased and ultimately (c) at high BSD, RMT was increasingly negatively correlated with EFS.

**FIGURE 10:**
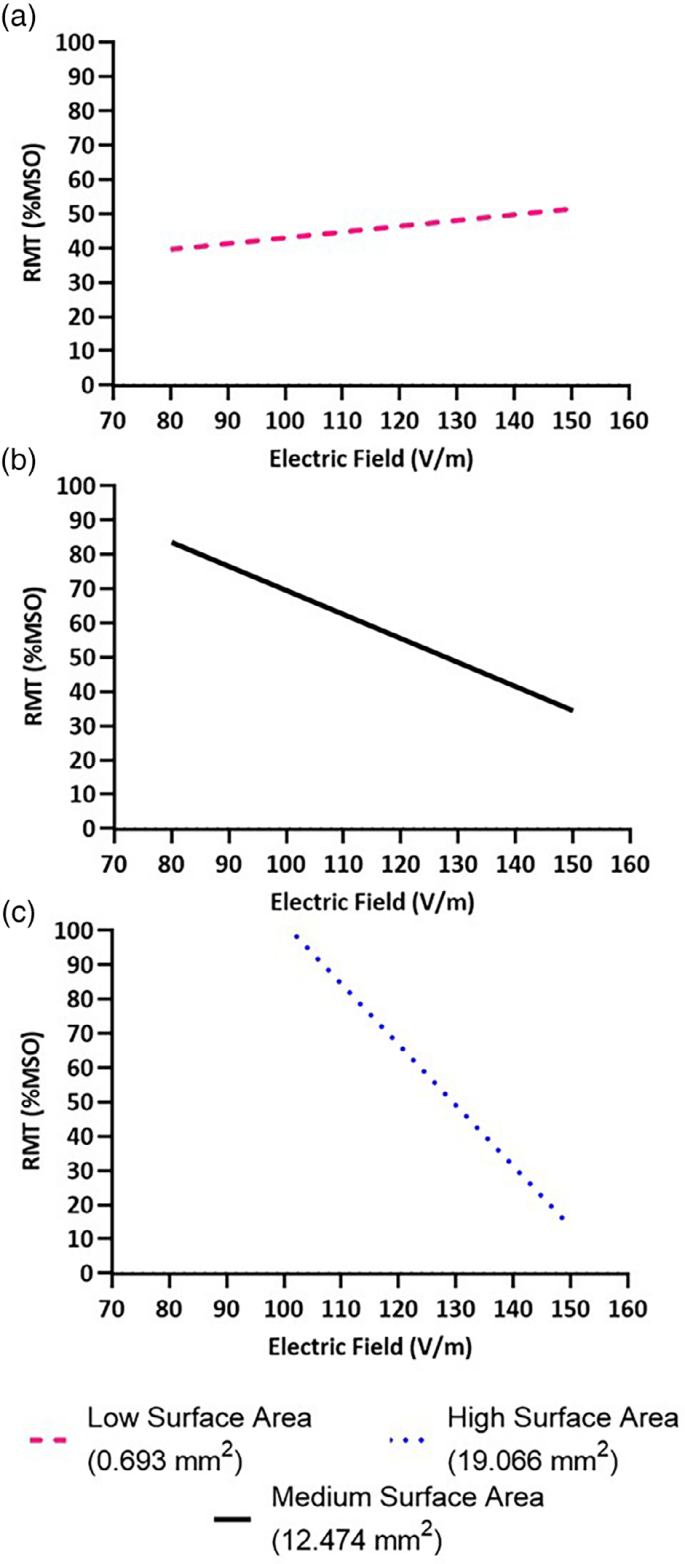
Effect of fiber tract surface area (FTSA) on empirically measured RMT of the biceps. (a) At lower FTSA, RMT was not strongly dependent on electric field strength (EFS). (b) As FTSA increased and ultimately (c) at high FTSA, RMT was increasingly negatively correlated with EFS.

The biceps RMT correlated with the interaction between EFS and FTSA. (*χ* ^2^ = 5.24, *p* = .022). The biceps RMT also correlated with the interaction between EFS and BSD (*χ* ^2^ = 6.68, *p* = .010), but there was no significant effect of TFC (*χ* ^2^ = 0.14, *p* = .712). Significant relationships are shown in Figures 9 and 10.

### 3.3 Effect of neuroanatomy on empirically measured change in nMEPs of the FDI after iTBS

**FIGURE 11:**
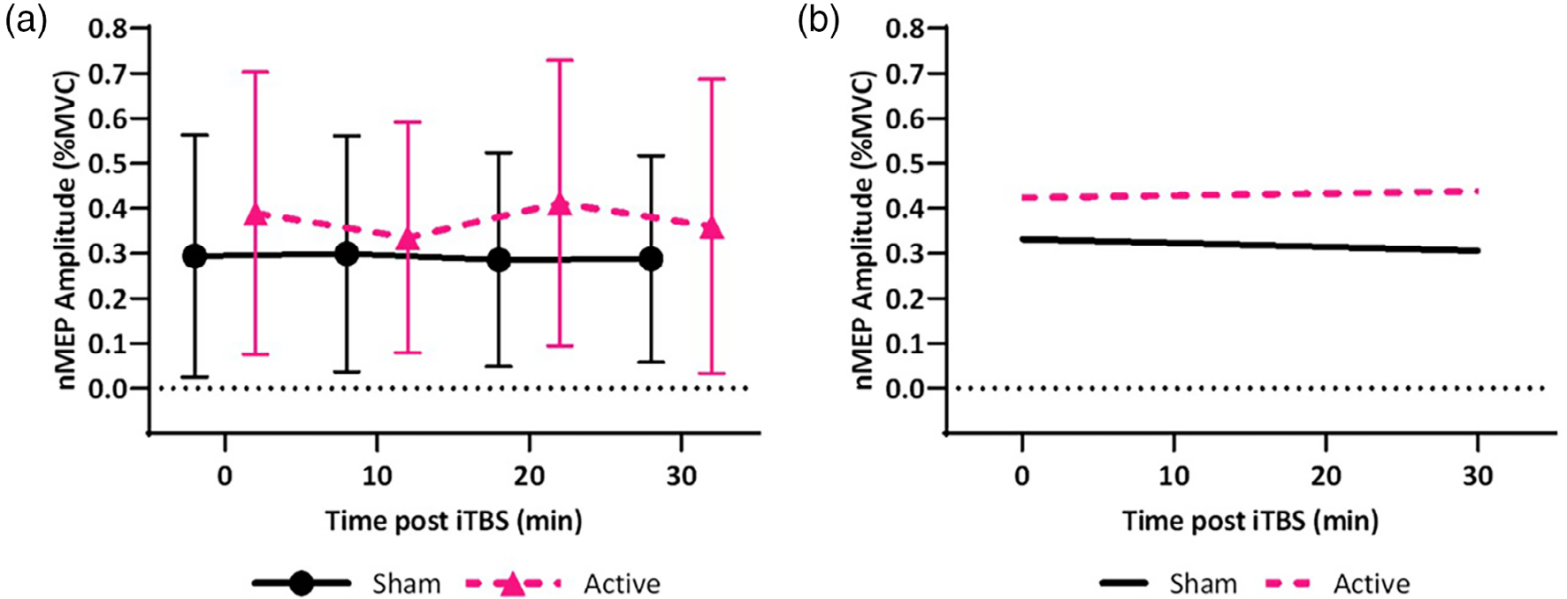
Effect of neuroanatomy on empirically measured change in nMEPs of the FDI after iTBS. (a) Mean FDI nMEP across the cohort is shown for each time point (error bars represent one standard deviation, 0 min represents the baseline). (b) Simulated nMEP are shown from the linear mixed effects model containing significant interactions between iTBS stimulation type (active or sham) and neuroanatomical parameters. nMEP amplitude after iTBS positively correlated with RMT, EFS, FTSA, and negatively with BSD.

There was no groupwide effect of iTBS on the FDI in this cohort (*χ* ^2^ = 1.48, *p* = .223) (Figure 11). Post‐iTBS change in FDI nMEPs correlated with the interaction between stimulation type (sham or active iTBS) and the following: RMT (*χ* ^2^ = 24.79, *p* < .001); EFS (*χ* ^2^ = 11.21, *p* = .001); BSD (*χ* ^2^ = 8.13, *p* = .004); and FTSA (*χ* ^2^ = 6.48, *p* = .011) (Figure 11). There was no effect of TFC (*χ* ^2^ = 1.26, *p* = .262).

### 3.4 Effect of neuroanatomy on empirically measured change in nMEPs of the biceps after iTBS

**FIGURE 12:**
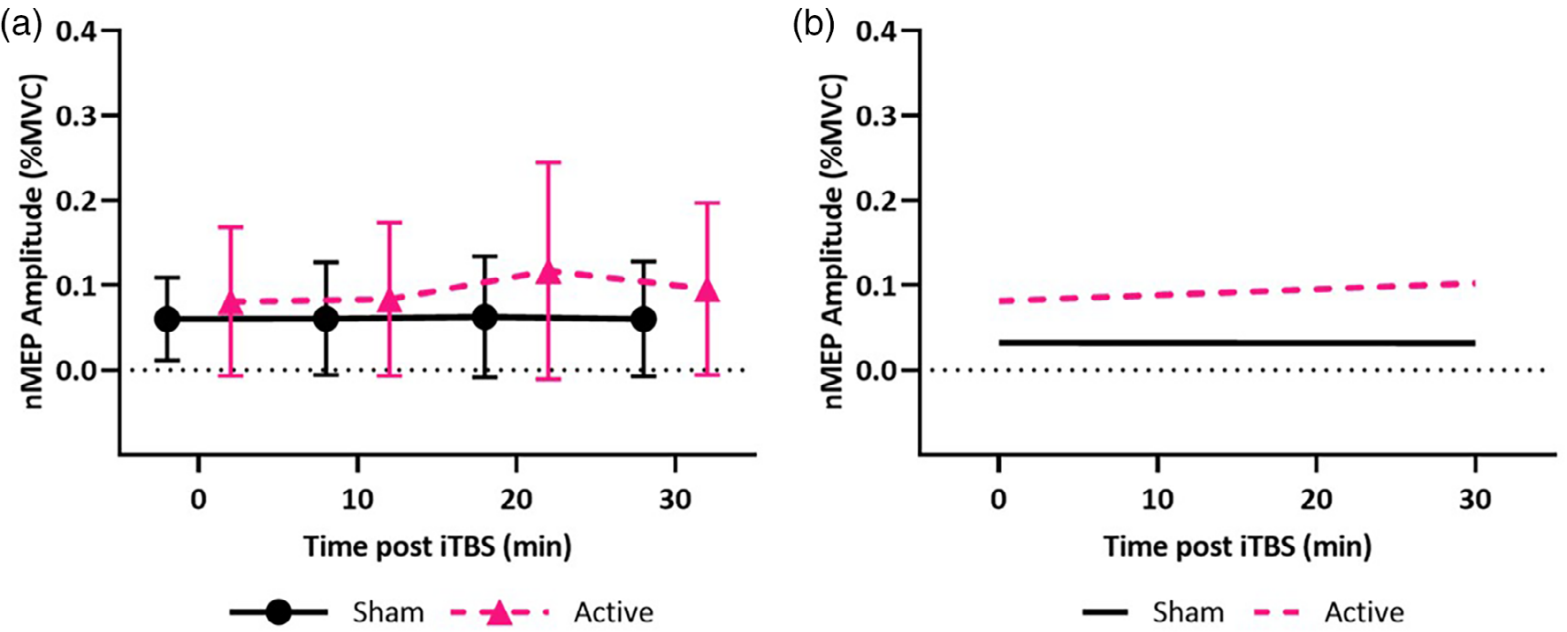
Effect of neuroanatomy on empirically measured change in nMEPs of the biceps after iTBS. (a) Mean biceps nMEP across the cohort is shown for each time point (error bars represent one standard deviation, 0 min represents the baseline). (b) Simulated nMEP are shown from the linear mixed effects model containing significant interactions between iTBS stimulation type (active or sham) and neuroanatomical parameters. nMEP amplitude after iTBS positively correlated with FTSA and TFC, and negatively with RMT.

iTBS had a facilitatory effect on the biceps (*χ* ^2^ = 6.12, *p* = .013) (Figure 12). Post‐iTBS change in biceps nMEPs correlated with the interaction between stimulation type (sham or active iTBS) and the following: RMT (*χ* ^2^ = 180.27, *p* < .001); FTSA (*χ* ^2^ = 19.11, *p* < .001); and TFC (*χ* ^2^ = 52.18, *p* < .001) (Figure 12). There was no effect of EFS (*χ* ^2^ = 0.08, *p* = .784) or BSD (*χ* ^2^ = 1.49, *p* = .223).

## 4 DISCUSSION

The aim of this preliminary study was to determine how individual neuroanatomy would affect the response of motor targets (FDI and biceps brachii) to single pulse TMS and iTBS paradigms. Identifying individual neuroanatomical characteristics that influence treatment response has the potential to inform future studies implementing iTBS techniques as a clinical treatment. Overall, response to both single pulse TMS and iTBS, of both the FDI and the biceps, depended on individualized neuroanatomical MRI‐derived parameters: EFS, FTSA, TFC, and BSD.

First, we hypothesized that RMT in the biceps and FDI would negatively correlate with the magnitude of the induced electric field, with fiber tract geometry affecting this relationship. This hypothesis was partially supported for both FDI and the biceps. The TMS response (RMT) of the FDI was dependent on fiber tract size and simulated induced electric field, whereas the biceps RMT was independent of fiber tract size. This suggests that tract organization plays a more important role than tract density in differentiating these two motor targets and their stimulation response to TMS.

The second hypothesis, that a greater field strength would correlate to a more excitatory effect of iTBS, was also partially supported. In the biceps, iTBS had a facilitatory effect, but this response was independent of induced electric field and BSD which represent cortical current. Instead, the excitatory effect of iTBS on the biceps correlated negatively with RMT, thus scaled with target sensitivity to TMS. Excitation was also greater in the presence of larger fiber tracts. In the FDI, however, there was no groupwide facilitation of iTBS. The FDI response was inversely related to the strength of current and correlated positively with higher RMTs, whereas higher induced electric field and greater TMS responsiveness correlated negatively with the iTBS response. Larger tracts and less stimulation attenuation also contributed to the iTBS changes for the FDI.

Our final hypothesis that specific factors such as electric field strength and fiber tract geometry would differently influence the response from the two motor targets (biceps and FDI) due to differences in cortical architecture, was also supported. The responses of the motor targets were variable, and response to TMS was not described sufficiently by BSD alone. iTBS of the biceps was more dependent on fiber tract parameters, while the smaller effect on the FDI was dependent on induced electric field. BSD was an influencing factor in some cases, negatively correlating with biceps RMT and with increase in corticomotor excitability after iTBS in the FDI, but less consistently so than the model‐derived parameters of electric field and fiber tract geometry. In the biceps, the effect of increasing BSD was to decrease RMT, contrary to expectation and to the electric field intensity, which highlights the limitations of using a single dimensional distance‐based metric to represent the complex tissue mediums through which the stimulation passes. Electric field intensity based on accurate head and brain anatomy provides further nuance for the calculation and therefore is more consistent with the empirical effect of TMS.

While the scope of this investigation did not include characterizing the effects of individual variability of the neuroanatomical parameters themselves, it is important to note that the relationships between TMS motor response and neuroanatomy were also variable and differed in some cases across the value of the neuroanatomical parameter. It stands to reason, as the measurements of TMS response, the physiological response itself to TMS, and neuroanatomy are all individually unique traits, that the relationships between these would also be complex and subject to fluctuations, represented by both intra‐ and inter‐individual variability. For example, despite BSD having limited utility in TMS response prediction relative to EFS, variability in BSD has an effect on EFS magnitudes (Knecht et al., 2005). Furthermore, neuroanatomical features that were not evaluated in this study could play a role in introducing more response diversity or relationship inversion, as electroelcephalogram (EEG) measurements can relate to maximal EFS specifically located at gyral crowns, uniquely from other cortical surface features (Bungert et al., 2017). Therefore, while directly explaining the drivers of the neuronatomy‐TMS response is beyond the scope of this study, future studies discerning the mechanisms underlying these relationships between TMS response, neuroanatomy, between neuroanatomical parameters, and even functional state of the brain regions (represented by fMRI blood‐oxygen‐level‐dependent images) would contribute to understanding the individual‐level differences in TMS response. Previously, relationships have been observed between DTI and RMT (Mirchandani et al., 2020). The novel contribution of the work presented here is the direct characterization of responses to TMS across different modalities (i.e., single pulse TMS and iTBS) and different targets of the motor cortex (i.e., control regions for the biceps brachii and the FDI), and their relationship to neuroanatomical measures.

These results suggest that MRI‐based measures of neuroanatomy have predictive value in the selection of TMS targets and confirm that cortical architecture is fundamentally influential in the motor system's response to neuromodulation paradigms. The parameters presented are predictive both in a homogeneously responsive group (biceps target) and a heterogeneously responsive group (FDI target) with regards to empirical response to both single pulse TMS and iTBS. This supports the proposition that individual anatomy plays a prominent role in TMS mechanisms.

The value of MRI‐based measures of neuroanatomy has numerous clinical implications. TMS and iTBS have seen increased use in recent years, especially in patients presenting with a variety of neurological and neuropsychiatric disorders (Anderson et al., 2020; Latorre et al., 2019). Our findings regarding the importance of individual neuroanatomy in treatment response address treatment response variability and provide a path forward for future work (Latorre et al., 2019). Neuroimaging techniques have been proposed (Herrold et al., 2020) to assist in the identification of motor targets or potential patients who, based on individual brain anatomy, are most likely to benefit from iTBS based therapies (Lee et al., 2016; Syeda, Magsood, et al., 2017). Diffusion‐weighted methods like DTI, provide an avenue through which to control for the individual differences that may underlie previous inconsistent evidence (Cash et al., 2020; Raffa et al., 2018; Sollmann et al., 2018). Although this work is a preliminary investigation, these findings support the use of neuroimaging‐derived techniques to inform the application of TMS treatment in clinical populations beyond the scope of neuronavigation. Furthermore, emerging neuroimaging techniques, such as neurite orientation dispersion and density imaging (NODDI), also have great potential to further address variability that may be arising from individual differences. NODDI is a relatively new in vivo diffusion MRI‐based analysis technique that allows for the estimation of microstructural complexity of dendrites and axons (Zhang et al., 2012). NODDI is one method for avoiding the well‐documented issues in diffusivity estimations that arise when DTI‐based estimation techniques are used on complex white matter structures (Bastiani et al., 2017; Zhang et al., 2012). Given our previously noted results indicating the importance of fiber tract count in RMT, it is possible that techniques (e.g., NODDI) that account for the microstructural complexity of white matter tracts could further shed light on this issue.

Other analysis techniques might also offer a unique perspective on the issue of individual variability in TMS treatment response. Fixel based analysis (FBA) is a new analytical technique that uses diffusion‐weighted MRI data to assess white matter micro‐ and macrostructure. FBA generates three primary metrics: fiber density (microstructure), fiber/bundle cross‐section (macrostructure), and a combination of the two (fiber density and fiber/bundle cross‐section) (Wallace et al., 2020). Given the similarity between FBA and the measures generated in the present work, it is worth investigating whether a FBA‐based pipeline would produce similar results to those reported here.

## 5 LIMITATIONS

This preliminary study included no clinical population as participants. The addition of clinical diagnoses to a proof‐of‐concept study, such as this, would make it difficult to assess whether findings were being driven by individual differences in neuroanatomy or by clinical disorders. Future work should consider applying these techniques in clinical populations. Regarding the TMS sessions, while the sham stimulation was delivered prior to active iTBS in each session to prevent any potential response to active iTBS from influencing the sham response, it is possible this decision resulted in an order effect. It is also possible that immediate effects of iTBS were not captured, as the first 10 min after stimulation were not evaluated. Post‐iTBS time points replicated previous work and were more focused on the time frame most realistic to application in rehabilitation protocols. These would take place most likely at least with some delay after iTBS priming (Hinder et al., 2014; Perellón‐Alfonso et al., 2018; Yamaguchi et al., 2018). The use of MEPs to capture changes due to iTBS could be considered a limitation, even though they remain a conventional approach to measuring corticomotor excitability at the time of stimulation. iTBS promotes long‐term potentiation of cortical neurons (Huang et al., 2005; Suppa et al., 2016), and multiple circuits contribute to individual MEPs making interpretation of changing amplitudes difficult (Bestmann & Krakauer, 2015). MEPs themselves exhibit high variability and represent a confounder in TMS studies (Kiers et al., 1993; Rösler et al., 2008), despite their utility and prevalence as a TMS metric. Future studies should look to TMS paired with electroencephalography (TMS‐EEG) to characterize instantaneous, neurophysiological sources of MEP variability. TMS‐EEG provides temporal and spatial resolution in the evaluation of interconnected neural networks at the time of TMS stimulation (Casula et al., 2021); associations between microstructural elements and TMS‐evoked EEG activity in participants post‐stroke were observed. Casula et al. similarly captured physiological inputs of other brain areas and how they affected motor control based on motor cortical synchronization as a response to motor TMS (Casula et al., 2018). Future studies should account for functional network connectivity, not just structurally, as was done with fiber tractography in the present study, but also with respect to motor cortical synchronicity and functional connectivity (Stephan & Friston, 2009), as could be observed by EEG and functional MRI, respectively. With respect to the simulations, the electric field was calculated using a magneto‐quasistatic solver to calculate what should be a time varying parameter in the finite element analysis. This simplification was made to account for reasonable approximation respective to available computing power. Furthermore, the simulated induced electric field was a single pulse TMS product, as we are currently unable to simulate time effects and repetitive stimulation so iTBS was not modeled. Future studies of iTBS responsiveness would benefit from simulations of cortical effects of iTBS stimulation itself. Finally, there are a number of contributors to variability that are not conventionally controlled, but based on the present study, may be used to normalize or control in future work. For example, EFS could be a normalization factor in order to improve comparisons of TMS outcomes and neuroanatomical effects between individuals, similar to the normalization of MEP measurements. EFS normalization has been proposed in psychology research (Turi et al., 2021), and may also improve consistency in motor TMS metrics. Alternatively, deep learning networks can be used non‐mechanistically to predict MEPs of FDI by using the obtained data from current study as training dataset. This work is currently in progress at author's laboratories.

## 6 CONCLUSION

This preliminary study evaluated the effects of individual neuroanatomy on RMT and corticomotor excitability after iTBS. Neuroimaging and modeling techniques were used to determine the brain scalp distance, simulated induced electrical field strength, fiber tract surface area, and tract fiber count, uniquely accurate to each participant. Our results demonstrate that these neuroanatomy‐based measures are predictive of RMT and iTBS outcomes for the biceps and FDI, albeit differently. One of the contributing factors to these differences could be the variability in the RMT and MEP data for the two muscles. Overall, individual anatomy is a driver of TMS response and MRI‐based modeling can be used to select responsive TMS motor targets based on brain scalp distance, electrical field strength, fiber tract surface area, and tract fiber count

## FUNDING INFORMATION

This study was supported by a research grant from the Virginia Commonwealth University Center for Rehabilitation Science and Engineering. Research operations were further supported by the Commonwealth Cyber Initiative (Proposal ID #: FP00010500) and the Virginia Commonwealth University Deans Undergraduate Research Initiative.

## CONFLICT OF INTEREST

Dr. Hadimani has one granted patent on a TMS coil (US15/795,057), two patents published and pending on TMS coils US15/335,286, US Patent Application 63,137,788), and another patent pending on an anatomically accurate brain phantom (US16/104,217).

## PARTICIPANT/PATIENT CONSENT

Written informed consent was obtained from all participants for participation.

## Data Availability

Data is available through the Open Science Framework—https://osf.io/qtxn4/?view_only=54a0e81eeb3e44f8a90971cbbd07c433.
